# *QuickStats*: Brain Cancer Death Rates Among Children and Teens Aged 1–19 Years,* by Sex^†^ and Age Group — United States, 2013–2015

**DOI:** 10.15585/mmwr.mm6617a5

**Published:** 2017-05-05

**Figure:**
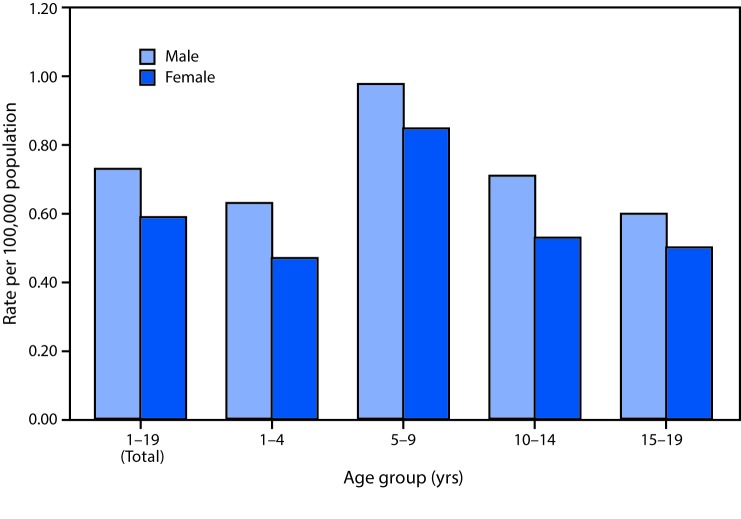
The death rate for brain cancer, the most common cancer cause of death for children and teens aged 1–19 years, was 24% higher in males (0.73 per 100,000) than females (0.59) aged 1–19 years during 2013–2015. Death rates were higher for males than females for all age groups, but the difference did not reach statistical significance for the age group 5–9 years. Death rates caused by brain cancer were highest at ages 5–9 years (0.98 for males and 0.85 for females).

**Sources:** National Vital Statistics System mortality data. https://www.cdc.gov/nchs/nvss/deaths.htm. NCHS data brief, no 257. https://www.cdc.gov/nchs/products/databriefs/db257.htm.

